# A Machine Learning Model Integrating Remote Sensing, Ground Station, and Geospatial Data to Predict Fine-Resolution Daily Air Temperature for Tuscany, Italy

**DOI:** 10.3390/rs17173052

**Published:** 2025-09-02

**Authors:** Giorgio Limoncella, Denise Feurer, Dominic Roye, Kees de Hoogh, Arturo de la Cruz, Antonio Gasparrini, Rochelle Schneider, Francesco Pirotti, Dolores Catelan, Massimo Stafoggia, Francesca de’Donato, Giulio Biscardi, Chiara Marzi, Michela Baccini, Francesco Sera

**Affiliations:** 1 Department of Statistics, Computer Science, Applications “G. Parenti”, University of Florence, 50134 Florence, Italy; 2 Unit of Biostatistics, Epidemiology and Public Health (UBEP), University of Padua, 35131 Padua, Italy; 3 Biological Mission of Galicia (MBG), Spanish Council for Scientific Research (CSIC), 15704 Santiago de Compostela, Spain; 4 Climate Research Foundation (FIC), 28003 Madrid, Spain; 5 Consorcio de Investigación Biomédica en Red de Epidemiología y Salud Pública (CIBERESP), 28029 Madrid, Spain; 6 Swiss Tropical and Public Health Institute (Swiss TPH), 4123 Allschwil, Switzerland; 7 University of Basel, 4003 Basel, Switzerland; 8 Environment & Health Modelling (EHM) Lab, London School of Hygiene & Tropical Medicine, London WC1E 7HT, UK; 9 ϕ-Lab, European Space Agency, 00044 Frascati, Italy; 10 Department of Land, Environment, Agriculture and Forestry (TESAF), University of Padua, 35020 Padua, Italy; 11 Interdepartmental Research Center of Geomatics (CIRGEO), University of Padua, 35020 Padua, Italy; 12 Department of Epidemiology, Lazio Regional Health Service, 00147 Rome, Italy

**Keywords:** air temperature, MODIS, Landsat 8, machine learning, remote sensing, urban heat island

## Abstract

Heat-related morbidity and mortality are increasing due to climate change, emphasizing the need to identify vulnerable areas and people exposed to extreme temperatures. To improve heat stress impact assessment, we developed a replicable machine learning model that integrates remote sensing, ground station, and geospatial data to estimate daily air temperature at a spatial resolution of 100 m *×* 100 m across the region of Tuscany, Italy. Using a two-stage approach, we first imputed missing land surface temperature data from MODIS using gradient-boosted trees and spatio-temporal predictors. Then, we modeled daily maximum and minimum air temperatures by incorporating monitoring station observations, satellite-derived data (MODIS, Landsat 8), topography, land cover, meteorological variables (ERA5-land), and vegetation indices (NDVI). The model achieved high predictive accuracy, with R^2^ values of 0.95 for Tmax and 0.92 for Tmin, and root mean square errors (RMSE) of 1.95 °C and 1.96 °C, respectively. It effectively captured both temporal (R^2^: 0.95; 0.94) and spatial (R^2^: 0.92; 0.72) temperature variations, allowing for the creation of high-resolution maps. These results highlight the potential of integrating Earth Observation and machine learning to generate high-resolution temperature maps, offering valuable insights for urban planning, climate adaptation, and epidemiological studies on heat-related health effects.

## 1 Introduction

The impacts of non-optimal temperatures on mortality and morbidity have been well established, with findings showing an increased risk at both high and low temperatures [1–3]. Non-optimal temperature is a leading health burden, which is projected to further increase with a warming planet [4–6]. The World Meteorological Organization confirmed 2024 to be the warmest year on record, with global temperatures measuring at 1.55 °C above pre-industrial levels [7]. Besides contributing to an intensification in natural disasters, the climate crisis has exacerbated extreme temperature events with severe heat days increasing in intensity, duration, and frequency.

Until recent years, environmental health studies investigating the association between temperature and human health have typically estimated exposures at the city or national level based on the air temperature (also known as ambient temperature or near-surface temperature) measured at one or a limited number of available monitoring stations [4]. Monitoring stations offer convenient access to continuous meteorological data, which makes them a commonly used source in large-scale epidemiological studies. Despite this advantage, reliance on data from these stations may introduce systematic biases, as such measurements often lack the spatial resolution necessary to capture fine-scale temperature variations. This limitation is further exacerbated by the fact that meteorological stations are often unevenly distributed across urban and regional areas and are commonly located outside densely populated zones (e.g., at airports). As a result, the representativeness of exposure estimates may be compromised, potentially leading to exposure misclassification [8].

In recent years, climate reanalysis products have been increasingly used as an alternative data source for comprehensive background information in climate conditions. These products are obtained by running a series of global or regional weather forecasting models under observationally constrained scenarios via data assimilation [9]. Compared to in situ measurements, climate reanalysis products offer the advantage of delivering consistent historical records of numerous meteorological variables at various spatial and temporal resolutions across the entire globe. These datasets show good validity on the estimates for health-temperature associations, allowing for the assessment of heat-related health impacts at country or global level [9,10]. However, these global models, such as the ECMWF ERA5-land with a resolution of 10 km *×* 10 km, are too coarse to capture intra-urban temperature variability.

This limitation is particularly relevant because urban areas frequently experience temperatures several degrees higher than surrounding rural areas, especially at night, primarily due to the built environment absorbing and storing heat during the day and releasing it at nighttime. This phenomenon, called the urban heat island (UHI), can lead to an average temperature difference between urban and rural areas of 2–4 °C [11]. Contributing factors include the prevalence of impervious surfaces, lack of vegetation, and anthropogenic heat emissions from transport systems and industry [12,13]. Also, the urban population tends to be more vulnerable to heat stress, due to factors such as population density and socio-economic inequalities [14]. In addition to global warming and synergistic effects with high air pollution, aging populations and increased urbanization are conducive to future susceptibility to non-optimal temperatures [11,15]. Assessing these vulnerabilities requires a detailed understanding of how temperature varies within cities, which is strongly influenced by the complex interactions between atmospheric dynamics and city-specific characteristics.

At finer spatial scales, such as microscale (<2 km) and building scale (<100 m), temperature exposure estimates have been derived using numerical or statistical models. Among the numerical approaches, the UrbClim model has been applied to analyze the urban UHI effect at a 100 m resolution in 100 European cities [16]. UrbClim is based on a soil–vegetation–atmosphere transfer scheme, extended to incorporate the physical properties of urban surfaces. Using this framework, Lauwaet et al. [16] generated hourly temperature data for a ten-year period (2008–2017). Numerical models can ensure physical consistency and allow for the evaluation of future scenarios or mitigation strategies; however, they require detailed input data and are computationally intensive, which may limit their application over very large areas or extremely long periods.

The alternative approach, statistical models, includes methods that calibrate land surface temperatures (LST) measurements with observations from monitors using a set of spatio-temporal predictors such as large-scale meteorological data and land use inventories. Spatially continuous observations of climate variables, especially LST, are available through satellite-based measurements. LST can be retrieved from polar-orbiting satellite platforms such as MODIS (Terra and Aqua) and Landsat. Despite offering superior spatial coverage and resolution, LST is less accurate than air temperature in representing the actual conditions to which individuals are exposed. Nevertheless, due to the strong correlation between the two, LST data are widely used to estimate daily air temperature. Statistical models are highly flexible, adaptable to different regions, and less computationally demanding than numerical models, and they can capture complex microclimatic patterns related to topography and intra-urban variability. However, their reliance on satellite and ground observations makes them sensitive to data gaps.

The first attempts of these methods used regression-based statistical models to obtain air temperature levels at up to 800 m resolution [17–21]. Recent studies used new approaches by applying machine learning methods to predict daily near-surface air temperature [22–31]. Two studies were even able to produce air temperature maps at a finer (200 m or 100 m) resolution [23,24].

Bussalleu et al. [31] produced Europe-wide daily 1 km *×* 1 km resolution models for mean, minimum, and maximum ambient temperature for the period of 2003–2020. Daily temperature maps for 6 years at a 1 km *×* 1 km resolution for Italy have been previously created for correlating vertical ground movements [32] and are available as open data [33]. In Tuscany, a high-resolution LST downscaling has also been applied specifically to the urban context of Florence [34].

This study aims to provide refined tools for the exposure assessment in studies investigating associations of short- and long-term exposure to temperature and health, and to better characterize areas more vulnerable to extreme temperatures and temperature variability in the Tuscany region. We aim to develop 100 m *×* 100 m resolution models of minimum and maximum ambient temperature in Tuscany for the year 2022. We used a two-stage machine learning framework that integrates remote sensing and ground station data and ensembles Extreme Gradient Boosting with Multivariate Adaptive Regression Splines, combined with high-resolution Landsat-derived LST and a wide set of spatial and spatio-temporal predictors, to enhance both temporal accuracy and spatial detail.

This work can help inform mitigation strategies and improve urban planning to reduce the exposure in cities and vulnerabilities in the population.

## 2 Materials and Methods

### 2.1 The Study Area

**Figure 1:**
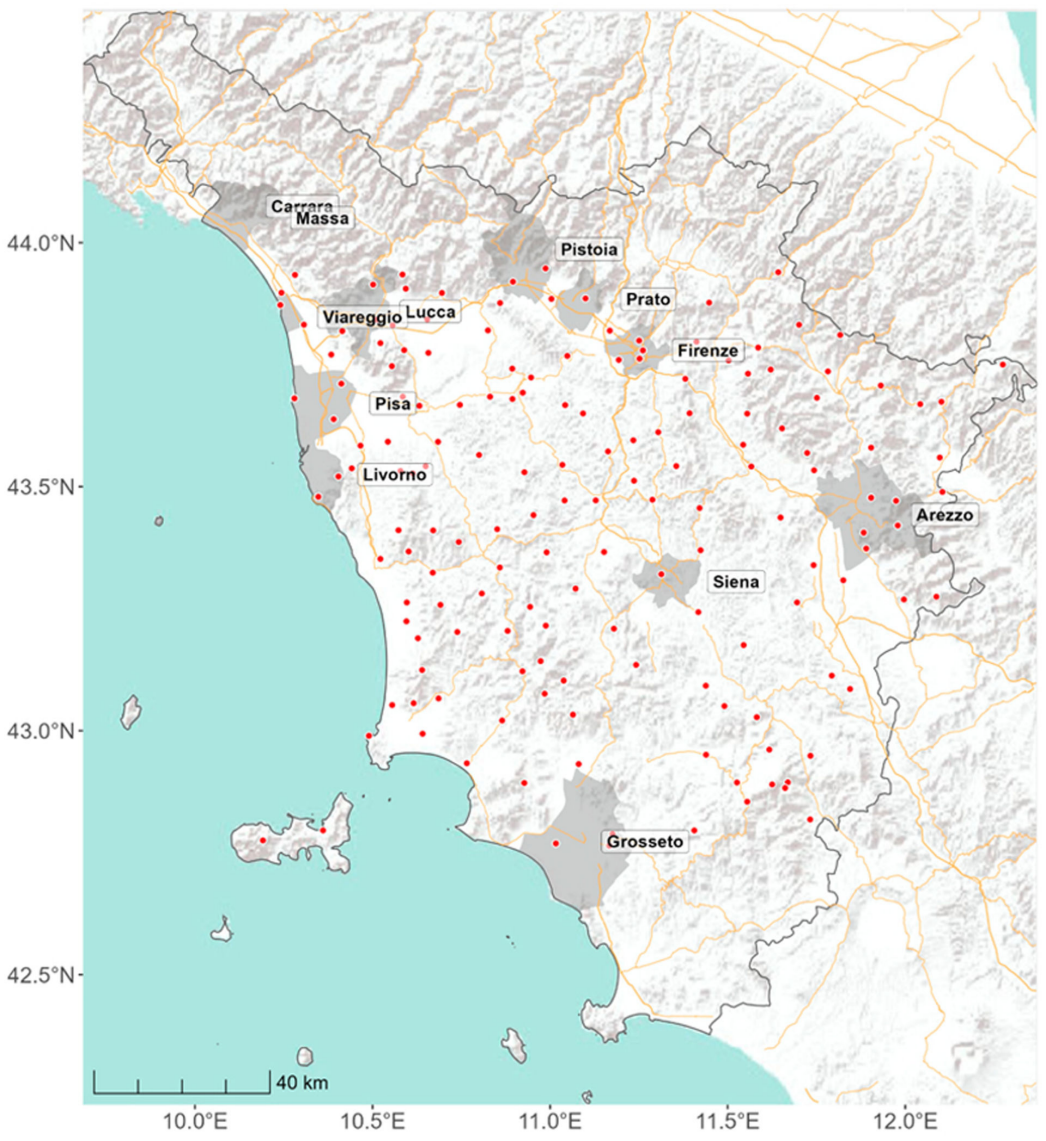
Distribution of the 162 active meteorological monitoring stations (red dots) in 2022 in Tuscany, Italy.

The study area consists of the entire territory of Tuscany (Figure 1), the fifth-largest region in Italy by surface area, encompassing approximately 22,993 km^2^. Tuscany features a remarkable variety of topographic characteristics, ranging from predominantly hills with about 66.5% of the total surface area, to 25% mountainous area and 8.5% plains [35]. The main mountain ranges include the Tuscan-Emilian Apennines in the northeast, with peaks exceeding 2000 m, such as Monte Cusna (2121 m), and the Apuan Alps in the northwest. Tuscany also features a coastal area of 633 km, including seven islands of the Tuscan archipelago [36]. The climate in Tuscany varies significantly due to its diverse topography. Coastal areas experience a Mediterranean climate, while the inland areas, particularly the hills and mountains, receive a more continental climate with colder winters and hotter summers. Tuscany had twelve cities with more than 50,000 inhabitants in 2022: Firenze (Florence), Prato, Livorno, Arezzo, Pisa, Pistoia, Lucca, Grosseto, Massa, Viareggio, Siena, and Carrara. These cities accounted for approximately 38.2% of the region’s population, while occupying 9.1% of its total land area [37].

Considering the Tuscany boundaries, we created a 100 m *×* 100 m grid, with a total of 2,306,665 cells (with irregular boundary cells following the boundaries of the polygon).

### 2.2 Data

#### 2.2.1 Meteorological Observations

The source of near-surface air temperature data was the database of the “Servizio Idrologico Regionale” (Regional Hydrological Service, SIR) [38]. This institution is responsible for the collection of quantitative meteorological–hydrological, groundwater, and tidal data through regional networks. The archived data undergo various quality check procedures before publication in the shared database. The current network consists of approximately 440 stations, 162 of them cover various areas of Tuscany (Figure 1) with meteorological data for the year 2022 [38]. Twenty-four of the 162 monitoring stations were classified as urban as they are located in the 12 cities with more than 50,000 inhabitants. For each monitoring station, we considered the daily minimum (Tmin) and maximum (Tmax) air temperatures. There were 4317 (7.3%) missing measurements for both Tmax and Tmin in a total of 54,813 daily measurements for all the monitors during the 365 days of 2022.

#### 2.2.2 Land Surface Temperature Data

We used version 6 of the Moderate Resolution Imaging Spectroradiometer (MODIS) daily 1 km land surface temperature (LST) and emissivity product from the Terra and Aqua satellites (MOD11A1 and MYD11A1, respectively). Each satellite provides a spatial resolution of 1 km *×* 1 km and orbits Tuscany twice per day, with overpass times at approximately 10:30 a.m. and 10:30 p.m. for Terra and 1:30 p.m. and 1:30 a.m. for Aqua (local solar time). Data for 2022 were obtained from the corresponding MODIS tile h18v04 from Google Earth Engine using the R library “rgee” (v1.1.3) [39,40]. Four variables were derived for each cell, representing values measured by MODIS during daytime (LST_ModisAD, LST_ModisTD) and nighttime (LST_ModisAN, LST_ModisTN) from the Aqua and Terra satellites. We used the quality assessment band to exclude pixels with an LST error of >2 K.

To characterize the seasonal spatial distribution of LST at a fine scale (100 m *×* 100 m) resolution, we used Landsat 8 satellite data from the United States Geological Survey [41]. Landsat 8 satellites have acquired images since 2013 with a frequency of 16 days. From the Google Earth Engine product “USGS Landsat 8 Level 2, Collection 2, Tier 1”, we used band 10 (ST_B10 surface temperature) to calculate LST with the formula LST = ST_B10 *×* 0.00341802 + 149 *−* 273.5, with a geometric resolution of 100 m *×* 100 m pixel size. Cloud mask and cloud filtering were implemented using the CFMASK algorithm, as well as a per-pixel saturation mask. Finally, for each season (winter, spring, summer, autumn), we composed all applicable LST retrievals. This yielded the LST_Landsat8 variable representing the median LST of each cell in each calendar season.

### 2.3 Spatial and Spatio-Temporal Predictors

We developed a harmonized geo-database combining Earth Observation (EO) satellite data and spatio-temporal predictors for Tuscany for the year 2022. The spatio-temporal predictors considered different characteristics associated with ambient temperature, including topography, sun geometry, meteorological variables, land cover, vegetation, population, and road network. The full list of predictors is reported in Table 1 with the temporal and spatial resolutions of the original data. The different features were available at different spatial resolutions ranging from 25 m *×* 25 m for topographic characteristics to 31 km *×* 31 km for planetary boundary height. For each day, the different products were harmonized into the Tuscany grid’s 100 m *×* 100 m resolution using area-weighted interpolation (function exact_extract of the R package exactextractr, v0.9.1) [42]. In the paragraphs below, each predictor is described in more detail.

We considered EU-DEM v1.0 (European digital elevation model) from the Copernicus Land Monitoring Service for elevation, slope, and aspect [43]. Slope identifies the steepest slope (in degrees) between the cell and its neighboring cells. Aspect depicts the downslope direction of the steepest slope (in degrees from 0 to 359.9, clockwise starting north). The sky view factor, a measure of the visible sky based on the digital terrain model, was calculated using the SAGA tool Sky View Factor in QGIS 3.4.4 [44–46]. Top-of-atmosphere diffusion and direct solar radiation, along with day length and sun altitude, were estimated for each grid cell using the “solrad” package in R (v1.0.0) [47]. The package uses day of the year, coordinates, slope, aspect, and elevation to estimate the potential diffusion and direct solar radiation in Watt per square meter, day length in hours, the solar azimuth angle, and solar altitude in degrees. Day length and solar azimuth angle were chosen as seasonal indicators.

Meteorological variables selected for the analysis included daily levels of relative humidity (2 m above the surface), 10 m horizontal wind speed and direction, total precipitation (Earth surface level), and surface pressure. We retrieved meteorological data from the Copernicus ERA-5 Land with a latitude–longitude grid size of 0.1° *×* 0.1°, roughly translating to a 9 km *×* 9 km grid [48]. Specifically, we extracted daily averages for temperature and dew temperature (2 m above the surface), 10 m U wind component, 10 m V wind component, surface pressure, and total precipitation. We calculated relative humidity (RH) from temperature and dew point temperature using the R “humidity” package (v0.1.5) [49] and the 10 m horizontal speed and direction from 10 m U and V wind components using the R “rWind” package (v1.1.7) [50,51].

We also considered the boundary layer height, which is the depth of air next to the Earth’s surface. This parameter is most affected by the resistance to the transfer of momentum, heat, or moisture across the surface. The boundary layer height can be as low as a few tens of meters for cooling air at night, or as high as several kilometers over the desert in the middle of a hot sunny day. The boundary layer height was obtained from ERA-5 with a latitude–longitude grid size of 0.25° *×* 0.25°, roughly translating to a 31 km *×* 31 km grid at the equator.

We considered impervious build-up (percentage share of build-up) as a contributor to anthropogenic heat. This indicator, at a resolution of 100 m *×* 100 m, was extracted from high-resolution layer (HRL) imperviousness data (for the year 2018) provided by the Copernicus Land Monitoring Service [52]. Land use data (also for the year 2018) were additionally extracted at a 100 m *×* 100 m resolution from the Corine Land Cover dataset provided by the Copernicus Land Monitoring Service [52]. The different land use categories were recoded into five main categories (Continuous_urban_fabric, Discontinuous_urban_fabric, Industrial_or_commercial_units, Vegetation, Agriculture).

To account for the spatial and temporal variation of vegetation, the normalized difference vegetation index (NDVI) was used as a proxy for greenness. The NDVI is measured daily by the MODIS instrument on board the Aqua and Terra satellites. The MODIS IV products (MOD13) are available at a 250 m *×* 250 m resolution. Monthly NDVI values were obtained from the MOD13Q1 V6.1 product for 2022 [53].

For the population and nighttime light, we used the 1 km *×* 1 km gridded population from Eurostat (JRC-GEOSTAT 2018) and the annual global Visible Infrared Imaging Radiometer Suite (VIIRS) Nighttime Lights (NTL) v2.2 dataset, provided by the Payne Institute for Public Policy at the Colorado School of Mines [54–56]. The NTL data for 2022 are available in raster format with a spatial resolution of 15 arc seconds (~500 m *×* 500 m at the equator) and represent nighttime light levels in nanowatts per square meter. The dataset containing the road network in Tuscany was sourced from the open data portal of the Tuscany Region [57]. The data, capturing road geometries and attributes, were provided in vector format and were last updated on 13 October 2022. The road network was used to characterize transportation infrastructure; for each cell, the length (in meters) of each road type (urban roads, local roads, urban secondary road, urban principal road, motorway and other roads) was calculated.

#### 2.4 Statistical Methods

**Figure 2:**
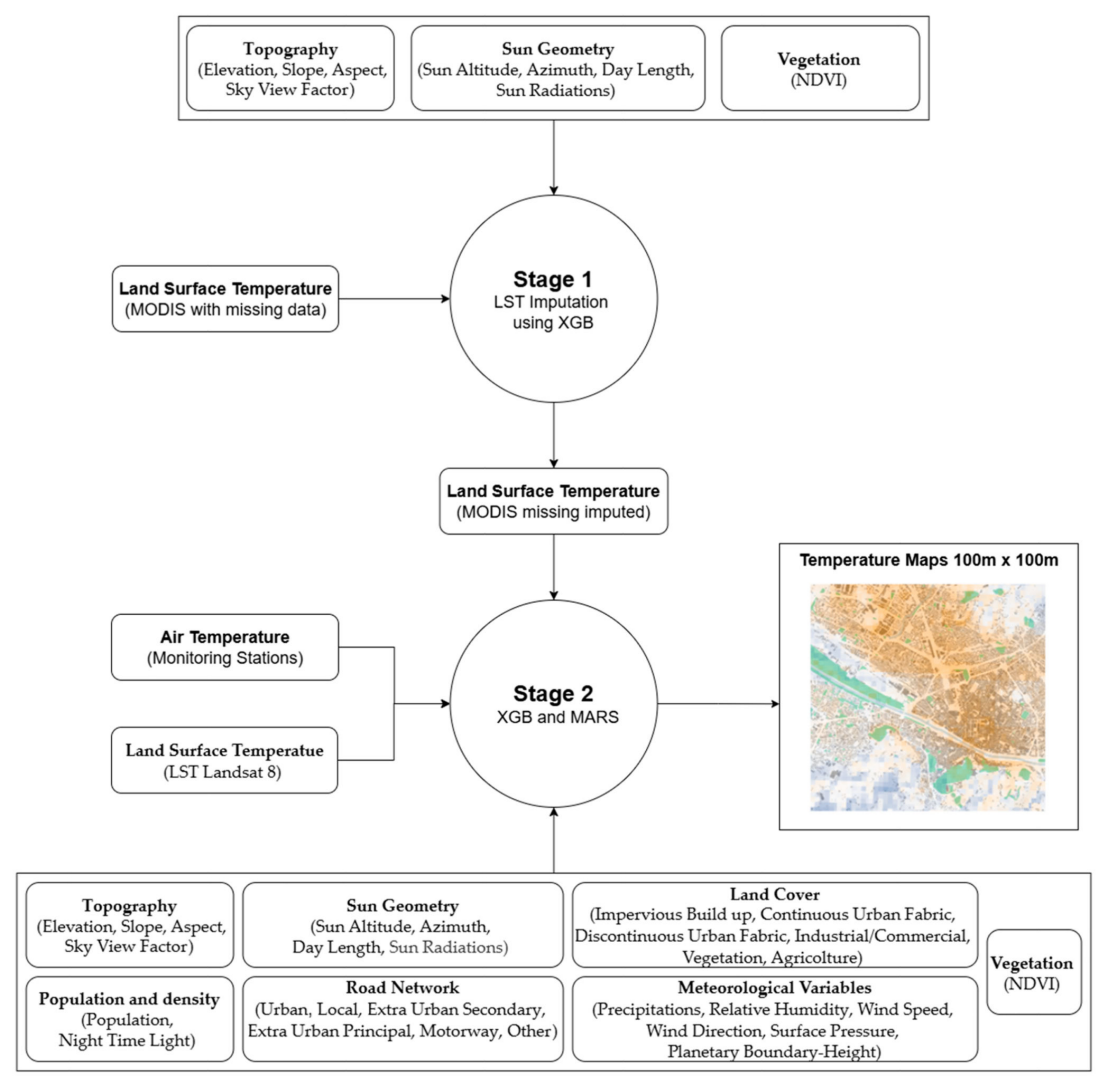
Two-stage modeling approach to estimate daily near-surface air temperature at a fine spatial resolution.

A two-stage modeling approach was used to estimate daily near-surface air temperature (Tmin, Tmax) at a fine spatial resolution (Figure 2).

In the first stage, we used the Extreme Gradient Boosting algorithm (XGB) for the imputation of missing MODIS (Terra and Aqua) satellite data. We imputed missing values by building four models for each day, using four variables (LST_ModisAD, LST_ModisTD, LST_ModisAN, LST_ModisTN), for grid cell *i* and day *j*, as described in Equation (1):

(1) $$\;LST\_Modis_{i,j}=\;XGB\;\left(\begin{array}{c} \;Elevation_{i,j},\;Slope_{i,j},\;Aspect_{i,j},\;Skyview_{i,j}\\ \;SunAltitude_{i,j},\;Azimuth_{i,j},\;DayLength_{i,j}\\ \;DiffuseSunRadiation_{i,j},\;DirectSunRadiation_{i,j},\\ \;NDVI_{i,j}, \end{array}\right)$$

We selected features related to topography (elevation, slope, aspect, sky view factor), seasonality (sun altitude, azimuth, day length, sun radiation), and vegetation (NDVI). We did not consider meteorological variables at this stage as they are related to the main outcome of the study, i.e., ambient temperature. After preliminary analysis, the XGB hyper-parameters were set as following: eta = 0.1, gamma = 0.01, min.child.weight = 100, max.depth = 10, subsample = 0.7, colsample_bytree = 0.7. The performance of the models was assessed using statistics based on out-of-bag (OOB) samples with a 5-fold cross-validation (CV) procedure. To this end, five random groups of observations were defined, and the complete outcome series in each group was predicted using a model fitted on the other four. Performance was evaluated using the R^2^, the root mean square error (RMSE), and the mean absolute error (MAE).

In the second stage, we applied the ensemble of two machine learning algorithms to predict the maximum and minimum ambient temperature for grid cell *i* and day *j*. In particular, we used the Extreme Gradient Boosting algorithm and the Multivariate Adaptive Regression Splines (MARS) model. The MARS model implements automatic selections (e.g., backward or forward) of non-parametric terms (e.g., splines) and their interaction [58].

XGB and MARS models, predicting daily ambient temperature for 2022, were separately developed for Tmin and Tmax using the predictors in Equation (2), as for example for Tmax:

(2) $$T_{m_{i,j}}=f\left(\begin{array}{c} \;Elevation_{i,j},\;Slope_{i,j},\;Aspect_{i,j},\;Skyview_{i,j},\\ \;UrbanRoad_{i,j}\;LocalRoad_{i,j},\;ExtraUrbanSecondaryRoad_{i,j},\\ \;ExtraUrbanPrincipalRoad_{i,j},\;Motorway_{i,j}\;OtherRoad_{i,j}\\ \;ImperviousBuildup_{i,j},\;ContinuosUrbanFabric_{i,j},\;DiscontinuousUrbanFabric_{i,j}\\ \;Industrial/Commercial_{i,j},\;Vegetation_{i,j},\;Agricolture_{i,j}\\ \;Population_{i,j},\;NightTimeLight_{i,j},\;DayLength_{i,j},\\ \;Precipitations_{i,j},\;RelativeHumidity_{i,j},\;WindSpeed_{i,j},\;WindDirection_{i,j},\\ \;SurfacePressure_{i,j},\;PlanetaryBoundaryHeight_{i,j},\;NDVI_{i,j},\\ \;LST\_ModisAD_{i,j},\;LST\_ModisTD_{i,j},\\ \;LST\_Landsats_{i,j}, \end{array}\right)$$

For Tmin, a similar set of predictors was chosen, but we considered variables derived from nighttime overpasses of the Terra and Aqua satellites: *LST*_*ModisAN*_*i*,*j*_, *LST*_*ModisTN*_*i*,*j*_.

The Extreme Gradient Boosting model parameters were set as follows: eta = 0.1, gamma = 0.01, min.child.weight = 100, max.depth = 10, subsample = 0.7, colsample_bytree = 0.7. For the MARS models, we considered a linear spline parametrization with one internal knot and no interaction with a stepwise forward selection based on 5-fold cross-validation procedure. We further considered an ensemble model averaging the predicted values using the XGB and MARS algorithms.

Similar to the first stage, the performance of the models and their ensemble combination in the second stage was assessed using statistics based on out-of-bag (OOB) samples with a 5-fold cross-validation (CV) procedure based on monitoring stations. Five random groups of locations with monitoring stations were defined, and the complete outcome series in each group were predicted using a model fitted using data from the monitoring stations in the remaining four groups. This validation procedure offers a measure of the true predictive ability of the models in locations where no ground data are available. Measures of performance were generated using predicted values on the observed series left out in each of the five runs, and computing the R^2^, root mean square error (RMSE), and mean absolute error (MAE). These statistics were computed using the whole set and then separated into spatial and temporal contributions. The former was computed using the averages of predicted and observed values across the entire series and offers a measure of performance in capturing long-term average ambient temperature values. The latter was computed as daily deviations from the averages and quantified the temporal variability explained by the model. Measures of performance of the ensemble model were also calculated for the four seasons and in the areas covered (urban) or not covered (non-urban) by the 12 biggest cities.

Once we assessed the validity of the XGB and MARS models and their ensemble combination, they were applied for each day in 2022 considering all grid cells in Tuscany to obtain the predicted ambient temperature Tmin and Tmax values.

## 3 Results

### 3.1 Stage 1

**Figure 3:**
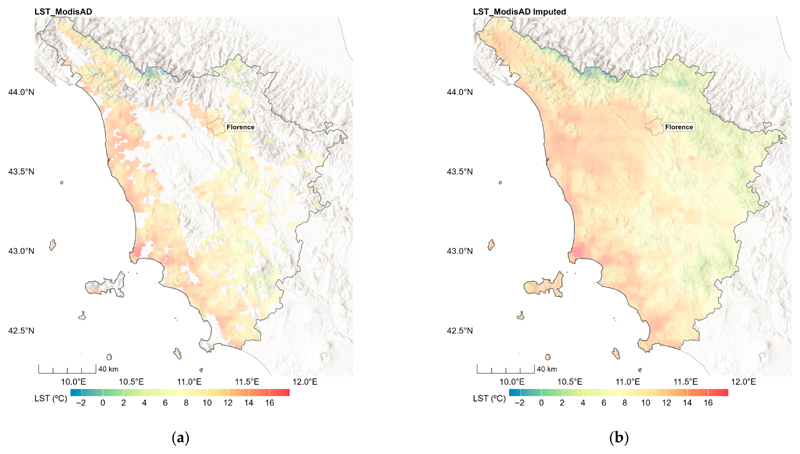
Daytime LST from Aqua MODIS before (**a**) and after (**b**) imputation on 1st March 2022 in Tuscany, Italy.

Table 2 shows the percentage of missing Modis-LST data for the different satellites, overpasses and seasons, which was mainly caused by cloud cover. The stage 1 XGB models explained large parts of the variation in the LST data (Table 2). The annual stage 1 models achieved an R^2^ over 0.99 and an RMSE between 0.13 °C and 0.46 °C. Figure 3 illustrates the LST_ModisAD data before (a) and after (b) stage 1 for the Aqua daytime overpass on 1 March 2022. The stage 1 model imputed the missing clear sky LST_ModisAD data, resulting in a complete “gap-filled” Aqua day.

### 3.2 Stage 2

**Figure 4:**
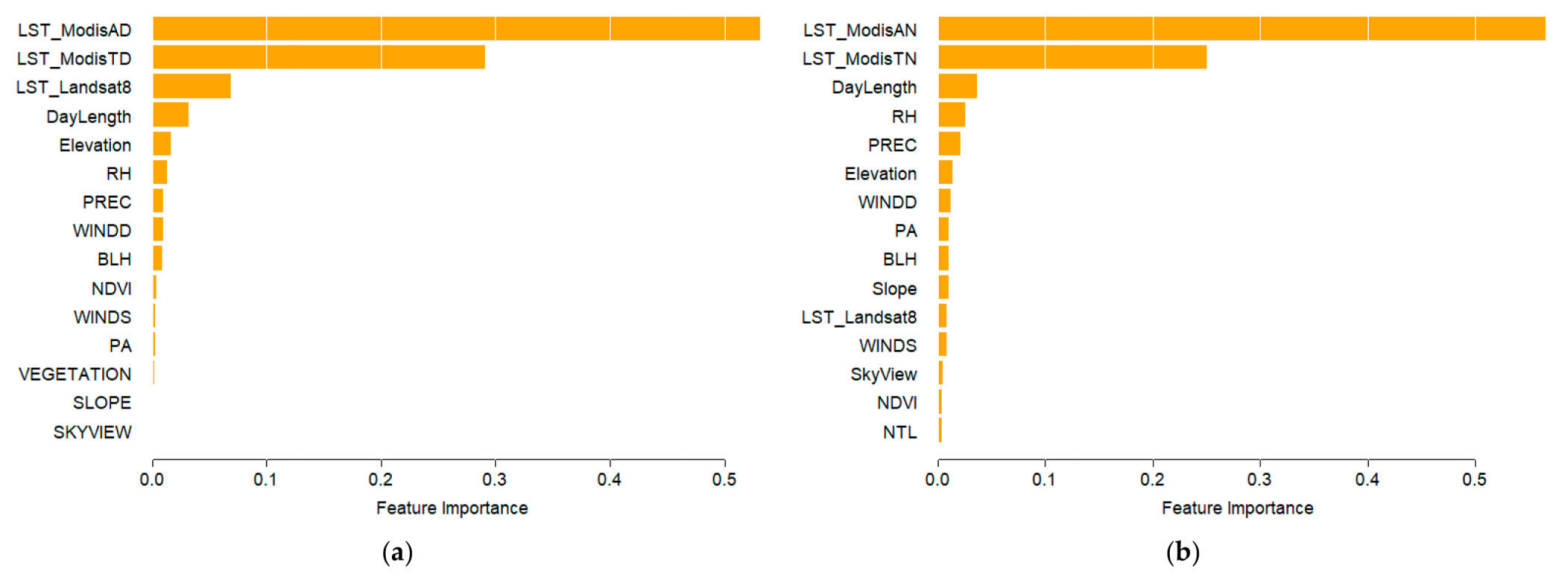
Feature importance (XGB) on modeling Tmax (**a**) and Tmin (**b**).

The feature importance of the Tmax and Tmin models from the XGB algorithm are shown in Figure 4. LST from MODIS was the most important predictor both for Tmax and Tmin. For Tmax, LST from Landsat was also an important predictor in addition to day length, meteorological variables, and topographic conditions. A similar pattern was observed for Tmin, for which LST from Landsat was a less important predictor. The only land use-related feature, modifiable by urban planning, was the NDVI, which ranked 8th for Tmax and 14th for Tmin.

**Figure 5:**
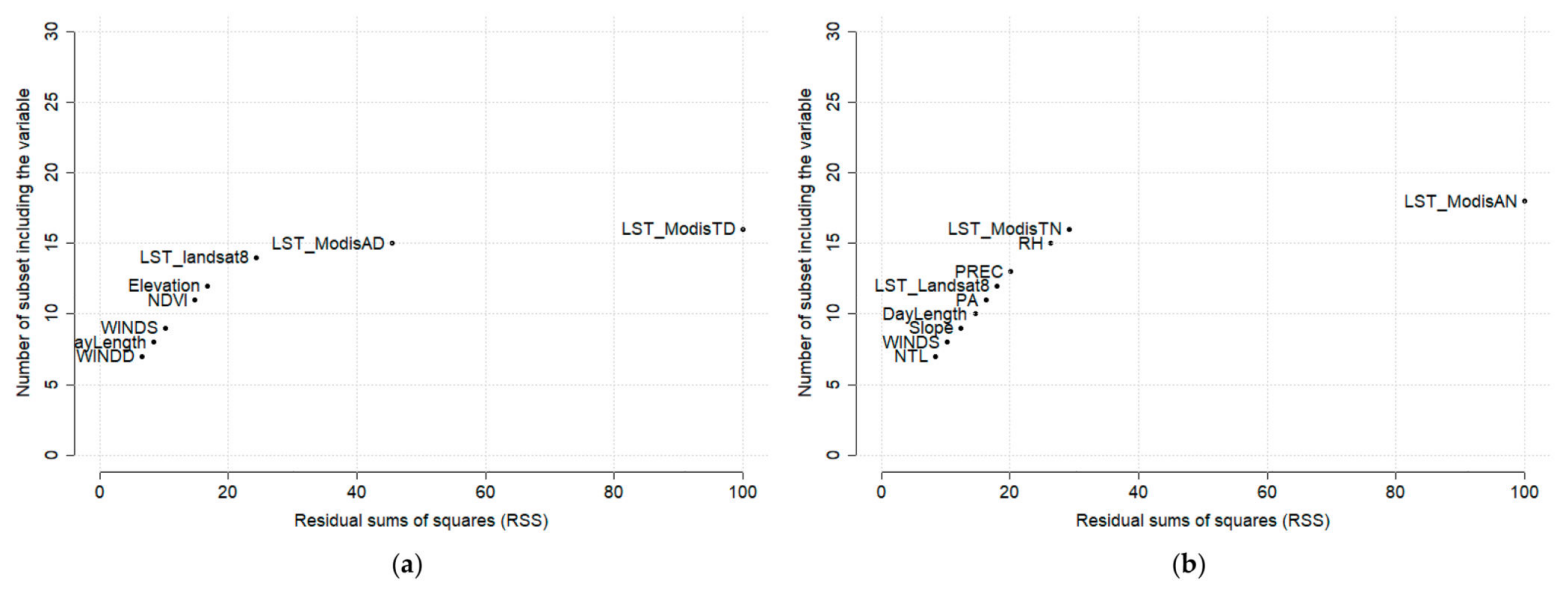
Feature importance (MARS) on modeling Tmax (**a**) and Tmin (**b**). On the x-axis, there is the residual sum of squares difference between the models containing and not containing the variable. On the y-axis, the number of “subsets” in which the variable is included is represented. A subset is a model with a smaller number of terms than that determined by the optimal model.

A complementary set of information was observed for the MARS model, where MODIS and Landsat measurements remained important predictors along with meteorological and topographic variables. However, vegetation as measured by NDVI, became a key predictor for Tmax, while nighttime light emerged as an important predictor for Tmin (Figure 5).

The validity (R^2^, RMSE, and MAE) of the stage 2 models on the hold-out validation set predicting daily Tmax and Tmin is shown in Table 3. For Tmax, the stage 2 model performed well, with an R^2^ and RMSE equal to 0.97 and 1.46 °C for the XGB algorithm and 0.88 and 2.99 °C for the MARS algorithm. Combining the information from the two models in the ensemble prediction yielded an R^2^ of 0.95 and an RMSE of 1.95 °C, with spatial and temporal R^2^ values of 0.92 and 0.95, respectively. For Tmin, the stage 2 model achieved similar performance, although with slightly lower accuracy: the XGB algorithm reached an R^2^ of 0.94 and an RMSE of 1.72 °C, while the MARS algorithm obtained an R^2^ of 0.86 and an RMSE of 2.56 °C. The two algorithms showed a similar spatial R^2^, while the XGB showed a higher temporal R^2^. Integrating the two models in the ensemble prediction resulted in an R^2^ of 0.92 and an RMSE of 1.96 °C, with a spatial and temporal R^2^ of 0.72 and 0.94, respectively.

Table 4 shows the validity measures of the ensemble models by season. For Tmax, a lower R^2^ and RMSE were observed in winter and summer, while for Tmin, winter and summer days were solely characterized by a lower R^2^.

The performance of the ensemble model for Tmax, assessed exclusively in the 12 biggest cities (urban areas), was comparable to those measured in the rest of Tuscany (non-urban area) (Table 5). For Tmin, a tendency toward higher R^2^ values was observed in urban areas.

**Figure 6:**
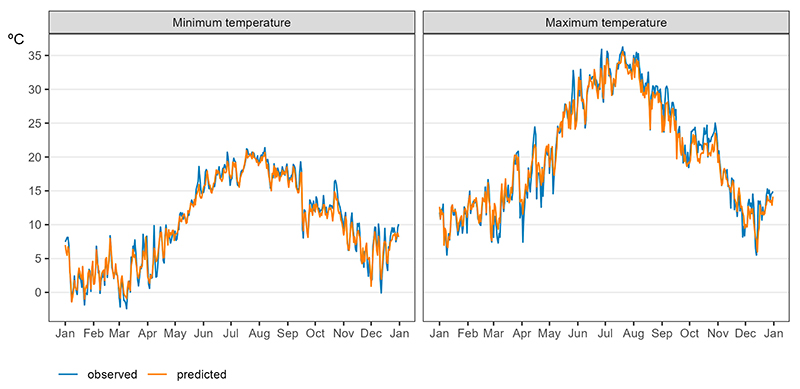
Predicted (orange) and observed by monitoring stations (blue) daily averaged Tmax and Tmin in Tuscany, Italy. Year 2022.

Figure 6 shows the predicted and observed daily Tmax and Tmin for the year 2022. Predicted daily values closely followed the measured observations. The mean difference between the observed and predicted daily ambient temperature values was 0.28 °C for Tmax and 0.19 °C for Tmin.

Figures S1 and S2 in the Supplementary Materials present boxplots of monthly ambient temperature Tmax and Tmin values, with lower average values in December and higher average values in July (Table 6).

**Figure 7:**
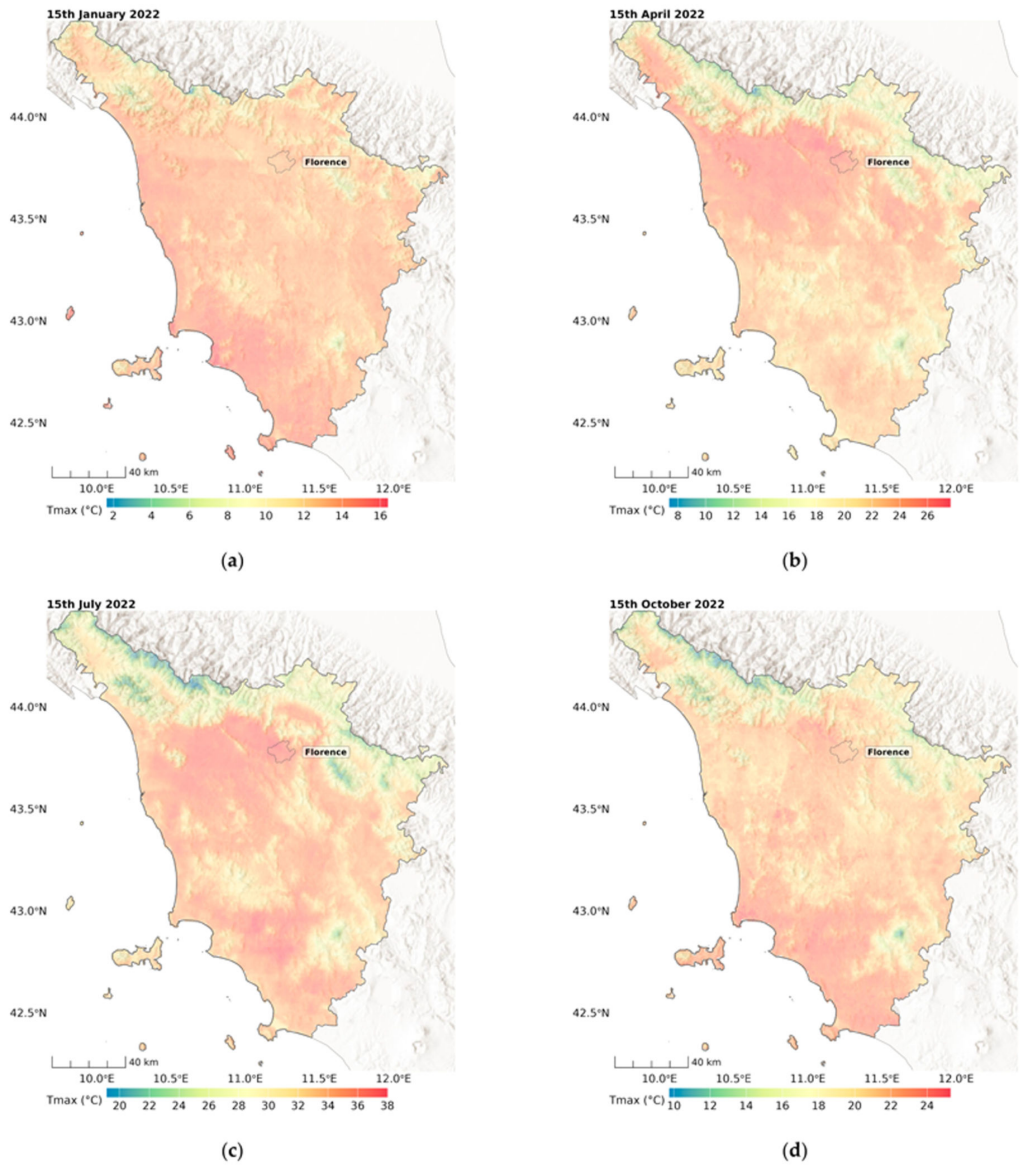
Predicted Tmax in days in the four different seasons: (**a**) winter, (**b**) spring, (**c**) summer, (**d**) autumn, for Tuscany, Italy.

**Figure 8:**
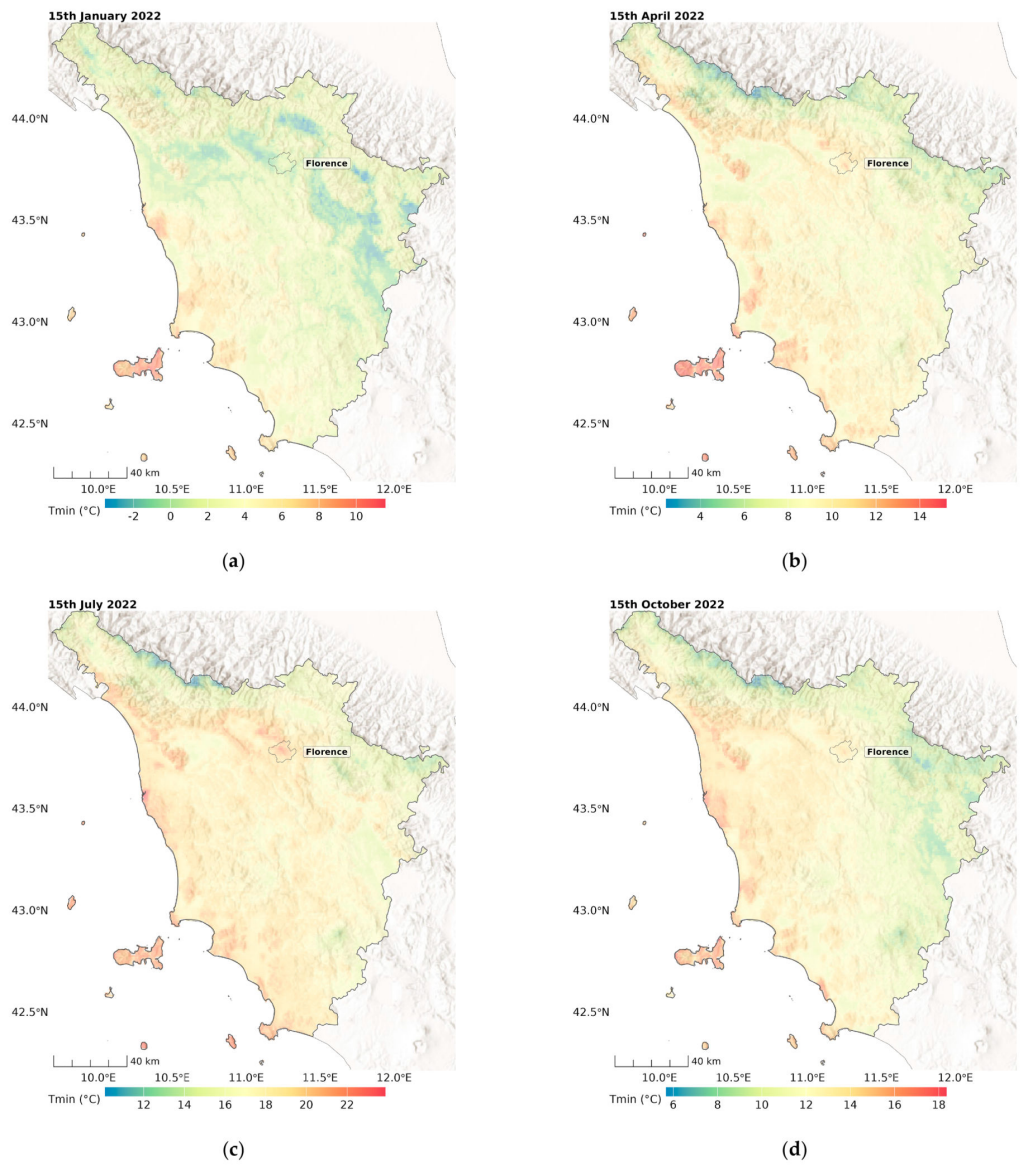
Predicted Tmin in days in the four different seasons: (**a**) winter, (**b**) spring, (**c**) summer, (**d**) autumn, for Tuscany, Italy.

Figures 7 and 8 show example maps of the predicted minimum and maximum temperature for all four seasons at a 100 m *×* 100 m resolution. As expected, more variability was observed in hotter seasons for Tmax, and higher temperatures were observed in the northwest regions, including the main cities of Florence and Pisa, and in the south in the Maremma plain land. Interestingly, some thermal inversion phenomena can be seen for Tmin in winter (15 January 2022), with lower temperatures in the east-oriented valley.

**Figure 9:**
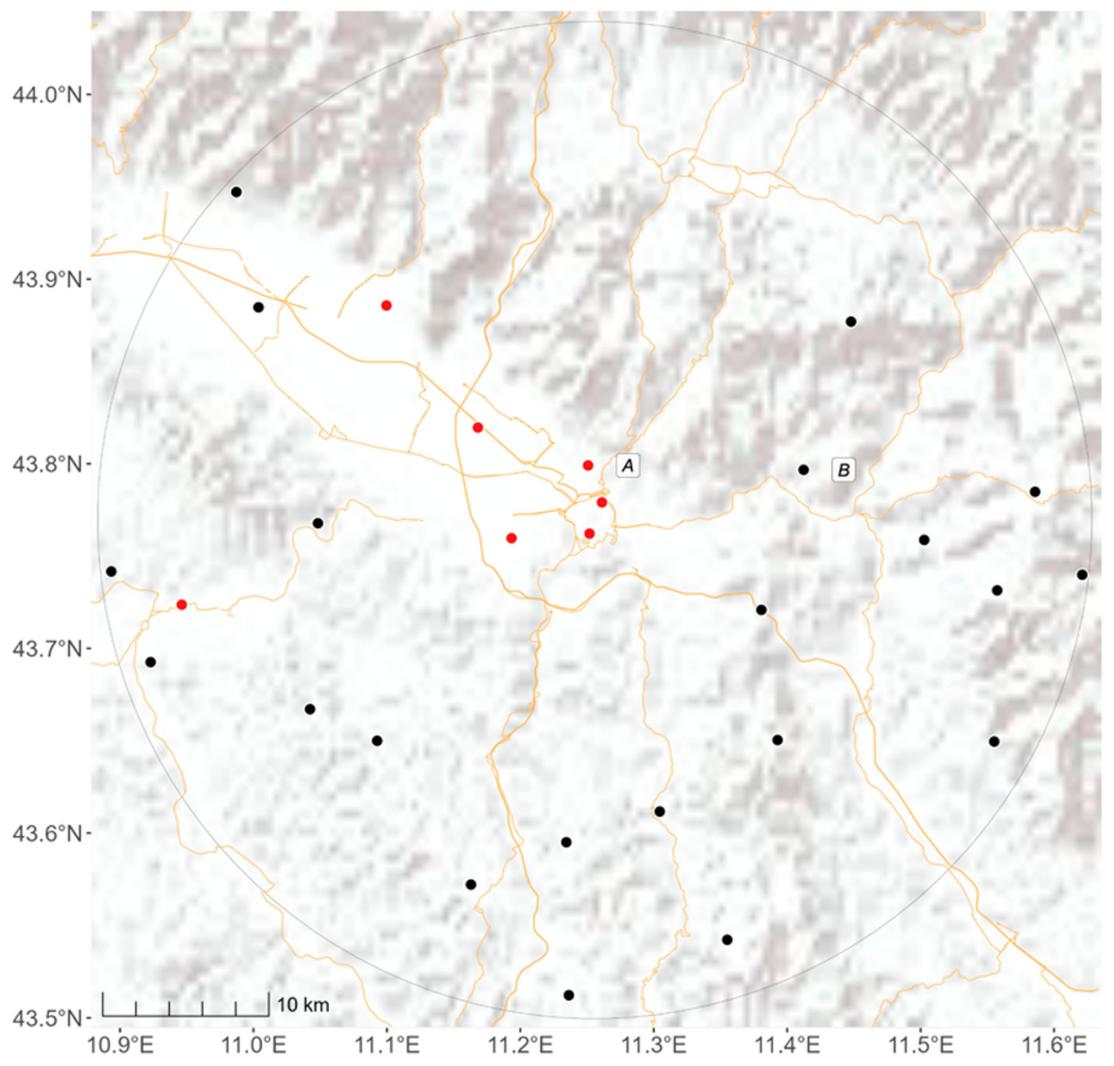
Placement of the 28 meteorological stations within the study area surrounding Florence (30 km circular buffer). Red dots represent urban stations and black dots represent non-urban stations. A corresponds to the University station in Florence and B to the station in Pontassieve.

For a more detailed analysis of the spatial distribution of the predicted temperatures, we performed a qualitative assessment considering the city of Florence and the surrounding area within a 30 km buffer as an example (Figure 9). There are 28 monitoring stations in the selected area: seven in an urban setting (red dots) and the others in non-urban settings (black dots), such as small villages, vegetation, or crop fields (Supplementary Table S1). The stations located in non-urban settings are located at a higher altitude than the urban stations (388 m versus 58 m). Comparing the observed maximum temperatures in the urban and non-urban stations, we detected a 2.37 °C difference. Our model estimated a similar difference of 2.34 °C with the predicted values. The difference is slightly higher than expected at 2.15 °C due to the altitude difference between cells containing urban and non-urban monitoring stations. Similar urban vs. non-urban differences (1.73 °C) were observed for the minimum temperature, while a difference of 1.85 °C was estimated with predicted model values.

For Tmax, the bias (difference between measured and predicted temperature values) was comparable between cells containing urban monitors (0.11 °C) and cells containing non-urban monitors (0.17 °C). Similar biases were estimated for Tmin, with a bias of 0.14 °C in cells containing urban monitors and a bias of 0.03 °C in cells containing non-urban monitors.

**Figure 10:**
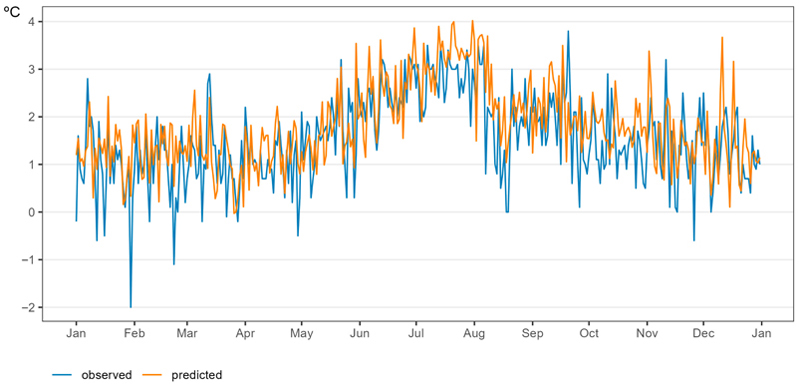
Observed (blue) and predicted (orange) differences in Tmin between urban and rural cells in the study area surrounding Florence over the year 2022.

Station B (Pontassieve) was chosen to represent a rural location near Florence. This station at an altitude of 230 m is surrounded by crop fields. For the urban reference, station A (Università), which is located near the university at an altitude of 80 m, was selected. The average daily observed difference between the urban and rural stations was 0.64 °C, which was comparable to the estimated 0.61 °C average daily difference calculated using predicted Tmax values, with a bias of 0.03 °C and an RMSE equal to 0.96 °C. This difference could be explained by the altitude difference between the two monitoring stations. Interestingly, a higher difference was observed for Tmin using observed values (1.54 °C) and predicted values (1.80 °C), with a bias of *−*0.26 °C and an RMSE equal to 0.77 °C. The seasonal difference was higher during warm months, suggesting a higher urban–rural difference during nighttime (Figure 10).

**Figure 11:**
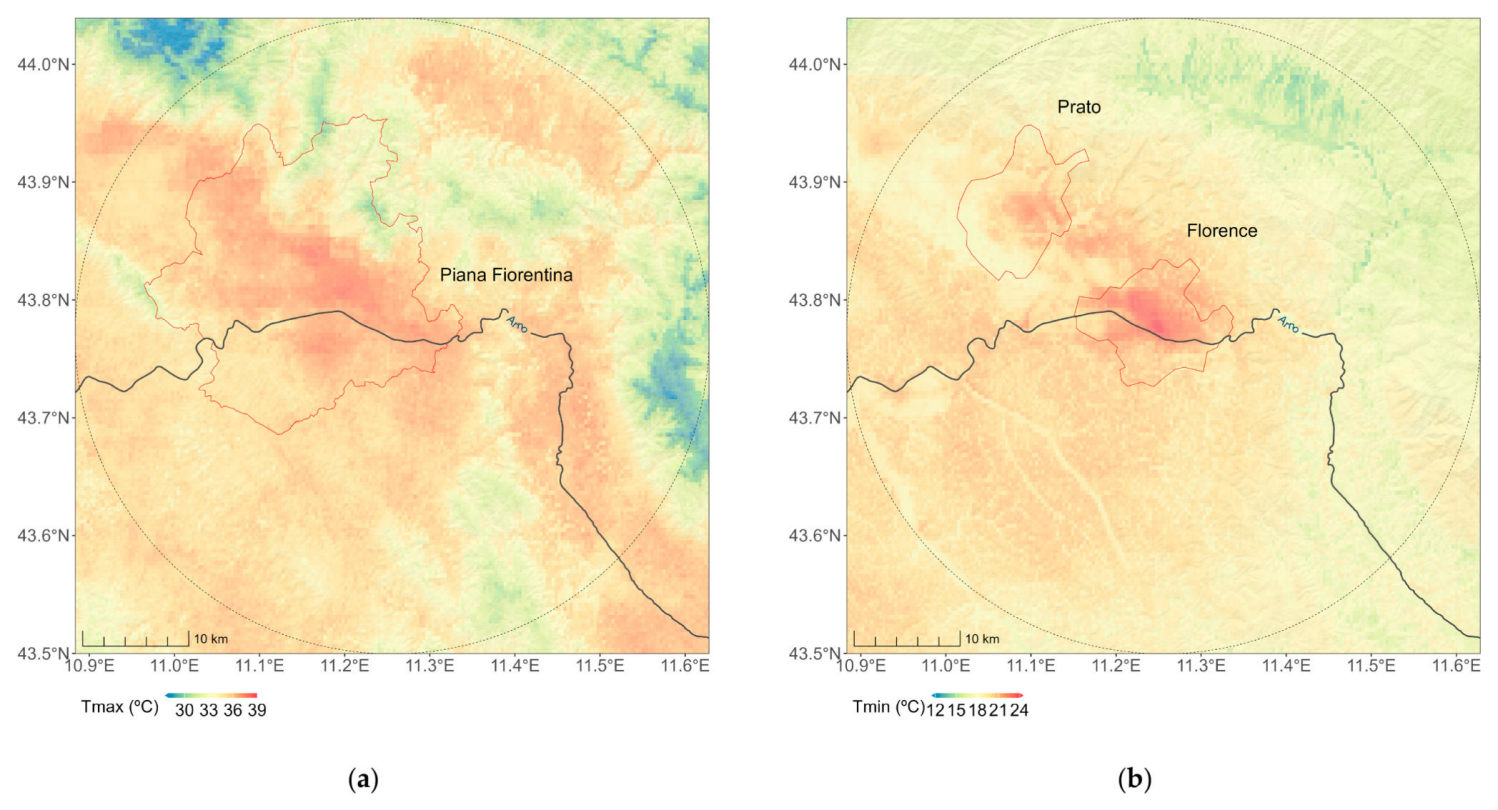
Predicted Tmax (**a**) and Tmin (**b**) in area surrounding (30 km) Florence (dashed circle), Italy (15 July 2022).

The predicted Tmax and Tmin values for one day (15 July 2022) in the study area are represented in Figure 11. The Tmax distribution is characterized by higher values in the “Piana Fiorentina”, an intermontane basin of alluvial origin with high level of urbanization, transport infrastructure, and economic activity encompassed by urban areas pertaining to the provinces of Firenze, Prato, and Pistoia in the heart of Tuscany’s largest metropolitan area. As expected, this distribution was similar (correlation coefficient of 0.72) to the distribution of the summer LST retrieved by Landsat (Supplementary Figure S3). The impact of urbanization was evident in the distribution of Tmin, with a hot spot in Florence and Prato.

## 4 Discussion

We developed temperature maps for the Tuscany region by integrating satellite data (MODIS and Landsat), topography, urban, and climate factors with local weather stations, and employing advanced machine learning techniques.

Several studies have provided high-spatial- and temporal-resolution maps of near-surface air temperature [16,24,59–68]. Most of them used numerical simulation models, such as MUKLIMO-3 [62,67], ENVI-met [59,60], COSMO [64], Weather Research and Forecasting (WRF) [63,65], and the ADMS-Urban model [61] to characterize and quantify the urban heat island effect. Given the high computational time, these simulations frequently estimate the urban temperature within a single city during a specific time period (e.g., during heat waves events) at a fine geographical scale (4.5 m to 300 m). Notably, within this class of numerical simulation models, Lauweat et al. [16] estimated hourly temperatures at a spatial scale of 100 m for 100 cities in Europe 2008–2017 using the UrbClim models. In recent years, statistical based models integrating remote sensing and monitor measurements with land cover and topographic spatio-temporal predictors were able to estimate daily air temperature at fine scale (10–30 m for the city of Oslo [68], 100 m for Switzerland [23] and 200 m for France [24]).

Our approach was built upon previous statistical-based models by mapping temperature at a high resolution of 100 m *×* 100 m. The main differences with those models are the broader spatial coverage with respect to Venter et al., which considers only the city of Oslo [68], and the finer spatial resolution with respect to Hough et al. [24]. In comparison with the model proposed by Flückiger et al. [23], we included Landsat as a predictor and considered an additional ML algorithm (MARS) to capture spatial heterogeneity. To our knowledge, no other study has produced temperature maps at this resolution for the Tuscany region. The methodology could be extended to produce high-resolution near-surface air temperature maps for other regions in Italy.

Building on the approach of previous studies, we relied on weather station networks to model temperature patterns. Hough et al. [24] and Flückiger et al. [23] achieved high accuracy in temperature mapping by combining MODIS data with weather station measurements. Similarly to Hough et al. [24], we integrated Landsat-derived land surface temperature at a 30 m *×* 30 m resolution. This finer granularity provides a more detailed representation of urban heat distribution by enhancing spatial detail and capturing intraurban variations more effectively than the 1 km *×* 1 km resolution of MODIS. The analysis of variable importance revealed that MODIS-derived LST was the most influential predictor for both maximum and minimum temperatures, which was also recognized as a key predictor by Flückiger et al. [23]. Landsat thermal data also played a significant role, but their influence was primarily limited to the estimation of maximum temperature. Topographic variables, such as elevation and solar geometry (e.g., day length) were additional factors that contributed to temperature modeling and have been widely used in past research to refine temperature estimates [25,26]. Meteorological conditions, including humidity and precipitation, were also integrated into the models, further supporting findings from prior studies [25]. The only land use-related feature that could be modified by urban planning is NDVI. This confirms the relationship between vegetation and temperature and the importance of considering the impact of green spaces on the temperature distribution when considering the relationship between temperature and health [69,70].

This study employed a hybrid modeling approach integrating machine learning with regression-based smoothing to enhance temperature prediction accuracy. Machine learning has been widely applied in temperature modeling, with Random Forest being the most frequently used method [23,25,27,28]. Some studies such as Zheng et al. [27] have explored alternative machine learning techniques, including histogram-based gradient boosting, extremely randomized trees, and deep belief networks. However, only one previous study incorporated XGBoost (XGB) within an ensemble framework [25]. To our knowledge, our study is the first to assess the performance of Multivariate Adaptive Regression Splines (MARS) for temperature modeling. The combination of XGB and MARS provides complementary advantages: XGB appears to better capture temporal structures by achieving a higher R^2^ in the temporal domain, while MARS captures more spatially related variability, with a higher spatial R^2^ especially for Tmax. Notably, this result was achieved without including temperatures from nearby stations. By integrating these two approaches, we aimed to enhance both the temporal consistency and the spatial granularity of temperature predictions.

In terms of model performance, our results showed an R^2^ of 0.95 for Tmax and 0.92 for Tmin, with corresponding RMSE values of 1.95 °C and 1.96 °C, respectively. These values are comparable to other studies in the literature. For instance, Flückiger et al. [23] reported an R^2^ between 0.94 and 0.99 and an RMSE ranging from 1.05 °C to 1.86 °C, while Hough et al. [24] achieved an R^2^ between 0.92 and 0.97 and an RMSE between 1.3 °C and 1.9 °C at a 1 km *×* 1 km resolution. Nikolaou et al. [26] reported an R^2^ between 0.91 and 0.96 with RMSE values ranging from 1.41 °C to 2.02 °C, whereas Jin et al. [25] obtained an R^2^ of 0.98 for an ensemble model with an RMSE of 1.38 °C. These comparisons indicate that our ensemble approach, combining XGBoost and MARS, performs at a level comparable to the best performing methods in the field, while offering a finer spatial resolution of 100 m × 100 m. In contrast with the work by Gutiérrez-Avila et al. [29], we observed a higher validity in temporal dimension compared to the spatial one. The R^2^ was 0.96 and 0.88 for Tmax and 0.95 and 0.72 for Tmin in the temporal and spatial dimensions, respectively. We observed a tendency for lower R^2^ in winter and summer days for both Tmax and Tmin. These results could be explained by narrower temperature ranges in winter and summer. In autumn and spring, higher temperature variability could influence (with higher values) the correlation coefficient (square root of R^2^). This interpretation is supported by similar if not lower values of RMSE and MAE in summer and winter days.

One of the main limitations of this study is the location of the monitor network not allowing for a quantitative assessment of the urban heat island effect. In each of the 12 large cities with more than 50,000 inhabitants, there are one to three monitoring stations. However, this monitoring network allowed us to gain some information on the spatial distribution of the temperatures predicted by our model. The results performed in the Florence area with 28 monitoring stations show that the spatial distribution of the maximum and minimum temperature follows an urban–rural pattern with higher air temperatures in urban areas. These observed urban–rural differences were similar to those estimated by our model, with a comparably low level of bias in urban and rural areas. There is a more evident temperature difference between urban and rural areas for the minimum temperature, with hot spots located in the cities of Florence and Prato.

An additional limitation of our study is the quality and reliability of satellite data. Cloud cover often obstructs land surface temperature retrieval, necessitating the use of imputation techniques [1]. While our approach leverages machine learning for data interpolation, uncertainties could remain, particularly in areas with persistent cloud cover. Additionally, the explicit consideration of temporal and spatial autocorrelation could further improve the model’s performance. Future work could explore the integration of these components into machine learning algorithms, for example, by using spatially aware models such as Random Forest with spatial and temporal lagged predictors. This may better account for temporal patterns, or considering feature extraction model based on graph neural network (GNN), namely the spatio-temporal estimation model (ST-GAT) recently proposed for PM_2.5_ concentration estimation [30]. Lastly, we did not specifically consider the uncertainty related to predictions that could be included in epidemiological research, but the validity measures we provided can be used to correct association measures [71].

Despite these limitations, the high-resolution temperature maps generated in this study offer valuable insights for multiple applications. First, they enable the precise delineation of urban heat islands, which is critical for urban planning and climate adaptation strategies. Second, the data facilitate the development of vulnerability indices, allowing policymakers to assess heat exposure risks more effectively. These maps can, for example, help analyze the impact of extreme heat events on vulnerable populations, particularly in densely populated cities. Furthermore, our results have direct applications in epidemiological research. In particular, the high validity in the temporal domain supports case cross-over or time series studies that investigate the short-term effects of temperature on health outcomes. In this context, while mortality remains the most extensively investigated health outcome linked to non-optimal temperatures [72–74], previous studies have also documented associations with a wide range of morbidity outcomes. These include increased risks of cardiovascular events such as myocardial infarction and heart failure [75–77], stroke, neurodegenerative diseases, mental health diseases [78], dehydration, respiratory conditions, and other heat-related illnesses [77]. Another important direction for future work would be the comparison of statistical and numerical models over the same areas, which could help validate both approaches and lead to more reliable applications in urban planning and health impact assessments.

## 5 Conclusions

This study presents a novel statistical modeling framework that integrates remote sensing and meteorological monitoring station data with land cover and topography information to predict high-spatial-resolution (100 m *×* 100 m) daily ambient Tmax and Tmin for 2022 in Tuscany. The statistical modeling framework was based on integrating two machine learning algorithms, XGB and MARS, and showed overall good performance under cross-validation strategies. By considering a spatial resolution of 100 m *×* 100 m, we were able to investigate the spatial distribution of Tmax and Tmin in a study area surrounding Florence. The predicted air temperature showed urban–rural differences, especially during nighttime, a pattern that could be explained by the urban heat island effect. This framework can be extended to other urban or non-urban regions in Italy. The modeled ambient temperatures can be used to describe the spatial distribution of near-surface air temperature and provide a valuable addition for epidemiological research investigating the health effects of heat.

## Supplementary Material

The following supporting information can be downloaded at: https://www.mdpi.com/article/10.3390/rs17173052/s1

Appendix

## Figures and Tables

**Table 1:** Spatial and spatio-temporal predictors included in the harmonized geocode database.

Dimension	Variable (Acronym)	Description	Unit of Measurement	Spatial or Spatio-Temporal	Original Spatial Resolution	Temporal Resolution	Stage
Topography	Elevation (DEM)	Digital Elevation Model	m	Spatial	25 m × 25 m	Constant (2016)	1;2
	Slope (SLP)	Steepest slope	Degree (angle)	Spatial	25 m × 25 m	Constant (2016)	1;2
	Aspect (Aspect)	Direction of the steepest slope, clockwise starting north	Degree (angle)	Spatial	25 m × 25 m	Constant (2016)	1;2
	Skyview (SVF)	Ratio of the visible sky (sky view factor)	Proportion	Spatial	25 m × 25 m	Constant (2016)	1;2
Sun geometry	SunAltitude (SUNALT)	Sun altitude	Degree	Spatio-temporal	100 m × 100 m	Daily (constant through years)	1
	Azimuth (Azimuth)	Azimuth	Degree	Spatio-temporal	100 m × 100 m	Daily (constant through years)	1
	DayLength (DAYL)	Day length	h	Spatio-temporal	100 m × 100 m	Daily (constant through years)	1;2
	DiffuseSunRadiation (DIFSUNRAD)	Diffuse solar radiation	$\frac{\text{W}}{{\text{m}}^{2}}$	Spatio-temporal	100 m × 100 m	Daily (constant through years)	1
	DirectSunRadiation (DIRSUNRAD)	Direct solar radiation	$\frac{\text{W}}{{\text{m}}^{2}}$	Spatio-temporal	100 m × 100 m	Daily (constant through years)	1
Meteorological Variables	Precipitations (PREC)	Total precipitation	m	Spatio-temporal	9km × 9 km	Daily	2
	RelativeHumidity (RH)	Relative humidity	Percentage	Spatio-temporal	9 km × 9 km	Daily	2
	WindSpeed (WINDS)	Wind speed	ms^−1^	Spatio-temporal	9 km × 9 km	Daily	2
	WindDirection (WINDD)	Wind direction	Degree (angle)	Spatio-temporal	9 km × 9 km	Daily	2
	SurfacePressure (PA)	Surface pressure	Pa	Spatio-temporal	9 km × 9 km	Daily	2
	PlanetaryBoundaryHeight (BLH)	Planetary boundary height	m	Spatio-temporal	31 km × 31 km	Daily	2
Land cover	ImperviousBuildup (IBU)	Impervious build-up	Proportion	Spatial	100 m × 100 m	Constant (2018)	2
	Continuous- UrbanFabric (CLC: Continuous Urban fabric)	Proportion of area covered by continuous urban fabric (from Corine Land Cover)	Proportion	Spatial	100 m × 100 m	Constant (2018)	2
	Discontinuous-UrbanFabric (CLC: Discontinuous Urban fabric)	Proportion of area covered by discontinuous urban fabric (from Corine Land Cover)	Proportion	Spatial	100 m × 100 m	Constant (2018)	2
	Industrial/Commercial (CLC: Industrial/ Commercial)	Proportion of area covered by industrial/commercial (from Corine Land Cover)	Proportion	Spatial	100 m × 100 m	Constant (2018)	2
	Vegetation (CLC: Vegetation)	Proportion of area covered by vegetation (from Corine Land Cover)	Proportion	Spatial	100 m × 100 m	Constant (2018)	2
	Agriculture (CLC: Agriculture)	Proportion of area covered by agriculture (from Corine Land Cover)	Proportion	Spatial	100 m × 100 m	Constant (2018)	2
NDVI	NDVI (NDVI)	Normalized difference vegetation index	Ratio (−1;1)	Spatio-temporal	250 m × 250 m	Every 16 days	1;2
Population and density	Population (POP)	Population	Persons/ Area	Spatial	1km × 1 km	Constant (2018)	2
	NightTimeLight	Nighttime light	$\frac{\text{nW}}{{\text{m}}^{2}}$	Spatial	15 arc seconds (~500 m × 500 m at the equator)	Constant (2022)	2
Road network	UrbanRoad (RDS: Urban Road)	Length of urban roads	m	Spatial	-	Constant (2020)	2
	LocalRoad (RDS: Local Road)	Length of local roads	m	Spatial	-	Constant (2020)	2
	ExtraUrbanSecondaryRoad (RDS: Extra UrbanSecondary Road)	Length of extra urban secondary road	m	Spatial	-	Constant (2020)	2
	ExtraUrbanPrincipalRoad (RDS: Extra UrbanPrincipal Road)	Length of extra urban principal road	m	Spatial	-	Constant (2020)	2
	Motorway (RDS: Motorway)	Length of motorway	m	Spatial	-	Constant (2020)	2
	OtherRoad (RDS: Other Road)	Length of other road	m	Spatial	-	Constant (2020)	2
Land surface temperature	LST_ModisAD	Land surface temperature from MODIS aqua day	K	Spatio-temporal	1 km × 1 km	Daily	2 Tmax
	LST_ModisTD	Land surface temperature from MODIS terra day	K	Spatio-temporal	1 km × 1 km	Daily	2 Tmax
	LST_ModisAN	Land surface temperature from MODIS aqua night	K	Spatio-temporal	1 km × 1 km	Daily	2 Tmin
	LST_ModisTN	Land surface temperature from MODIS terra night	K	Spatio-temporal	1 km × 1 km	Daily	2 Tmin
	LST_Landsat8	Land surface temperature from LANDSAT8	K	Spatio-temporal	30 m × 30 m	Every 16 days	2

**Table 2:** Performance measures of the stage 1 XGB models, by different satellites, overpasses, and seasons.

Variables	Observation Period	Number of Days with 100% NA	% NA	RMSE (°C)	R^2^	MAE (°C)
LST_ModisAD	All Year	35	76.8	0.317	0.992	0.221
	Winter	3	83.2	0.229	0.993	0.158
	Spring	24	84.6	0.344	0.992	0.241
	Summer	1	67.0	0.461	0.992	0.330
	Autumn	7	73.0	0.278	0.992	0.199
LST_ModisAN	All Year	42	78.9	0.162	0.994	0.113
	Winter	6	76.3	0.162	0.995	0.111
	Spring	25	90.2	0.173	0.994	0.120
	Summer	0	65.8	0.159	0.993	0.110
	Autumn	11	78.7	0.160	0.995	0.114
LST_ModisTD	All Year	33	81.4	0.250	0.994	0.174
	Winter	3	82.2	0.181	0.995	0.123
	Spring	6	82.5	0.285	0.993	0.201
	Summer	3	77.6	0.358	0.994	0.253
	Autumn	21	84.3	0.206	0.994	0.150
LST_ModisTN	All Year	44	82.1	0.148	0.995	0.101
	Winter	15	82.5	0.133	0.996	0.093
	Spring	15	87.0	0.147	0.995	0.102
	Summer	0	82.2	0.143	0.995	0.097
	Autumn	14	76.8	0.155	0.994	0.110

**Table 3:** Validation of performance of XGB, MARS and ensemble models in stage 2 for predicting Tmax and Tmin.

Variable	Model	RMSE (°C)	R^2^	MAE (°C)	Spatial R^2^	Temporal R^2^
Tmax	XGB	1.458	0.972	1.098	0.915	0.954
	MARS	2.991	0.881	2.329	0.906	0.880
	Ensemble	1.954	0.950	1.518	0.915	0.954
Tmin	XGB	1.715	0.938	1.314	0.715	0.941
	MARS	2.559	0.858	2.030	0.679	0.878
	Ensemble	1.961	0.920	1.530	0.715	0.941

**Table 4:** Performance measures of the stage 2 ensemble models in different seasons.

Variable	Season	RMSE (°C)	R^2^	MAE (°C)
Tmax	Winter	1.704	0.752	1.274
	Spring	2.306	0.896	1.839
	Summer	1.790	0.769	1.401
	Autumn	1.958	0.908	1.561
Tmin	Winter	2.024	0.757	1.592
	Spring	2.187	0.850	1.719
	Summer	1.723	0.647	1.350
	Autumn	1.883	0.845	1.465

**Table 5:** Performance measures of the stage 2 ensemble models in urban and non-urban areas.

Variable	Area	RMSE (°C)	R^2^	MAE (°C)
Tmax	Urban	2.001	0.944	1.577
	Non-urban	1.938	0.952	1.504
Tmin	Urban	1.863	0.935	1.464
	Non-urban	1.959	0.919	1.528

**Table 6:** Average predicted monthly ambient temperature Tmax and Tmin.

Month	Tmax (°C)	Tmin (° C)
January	10.6	2.4
February	12.6	3.4
March	14.6	2.9
April	17.9	6.3
May	23.9	12.1
June	30.1	16.9
July	32.8	18.8
August	30.0	18.1
September	23.4	14.0
October	21.2	12.1
November	15.3	7.3
December	11.4	6.5

## Data Availability

Data and programs are available under request to first and last author.

## Abbreviations

AD: Aqua Day
AN: Aqua Night
BLH: Planetary Boundary Layer Height
CLC: Corine Land Cover
CV: Cross-Validation
DAYL: Day Length
DEM: Digital Elevation Model
DIFSUNRAD: Diffuse Solar Radiation
DIRSUNRAD: Direct Solar Radiation
EO: Earth Observation
GNN: Graph Neural Network
HHAP: Heat Health Action Plans
IBU: Impervious Build-up
LST: Land Surface Temperature
MAE: Mean Absolute Error
MARS: Multivariate Adaptive Regression Splines
MODIS: Moderate Resolution Imaging Spectroradiometer
NDVI: Normalized Difference Vegetation Index
NTL: Nighttime Light
OOB: Out-of-Bag
PA: Surface Pressure
PM2.5: Particulate Matter < 2.5 µm
POP: Population
PREC: Total Precipitation
RDS: Road Network Dataset
RH: Relative Humidity
RMSE: Root Mean Square Error
SLP: Slope
ST-GAT: Spatio-Temporal Graph Attention Network
SUNALT: Sun Altitude
SVF: Sky View Factor
TD: Terra Day
Tmax: Maximum Temperature
Tmin: Minimum Temperature
TN: Terra Night
UHI: Urban Heat Island
WINDD: Wind Direction
WINDS: Wind Speed
XGB: Extreme Gradient Boosting
