# Supplementary material for: A Machine Learning Model Integrating Remote Sensing, Ground Station, and Geospatial Data to Predict Fine-Resolution Daily Air Temperature for Tuscany, Italy
Source: Remote Sens (Basel). Author manuscript; Available in PMC 2025 Sep 13. (PMC7618111; doi:10.3390/rs17173052)
Supplement: Appendix [file EMS208663-supplement-Appendix.pdf]

Figure S1. Tmax by Month, Tuscany, Italy. Year 2022.

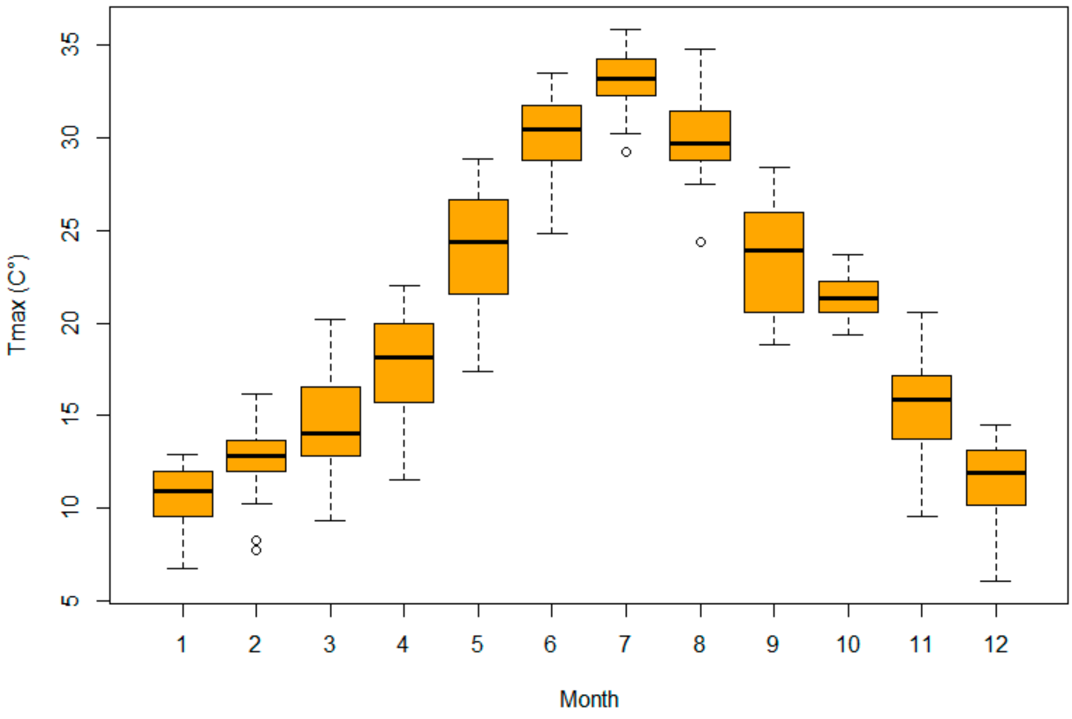

Figure S2. Tmin by Month, Tuscany, Italy. Year 2022.

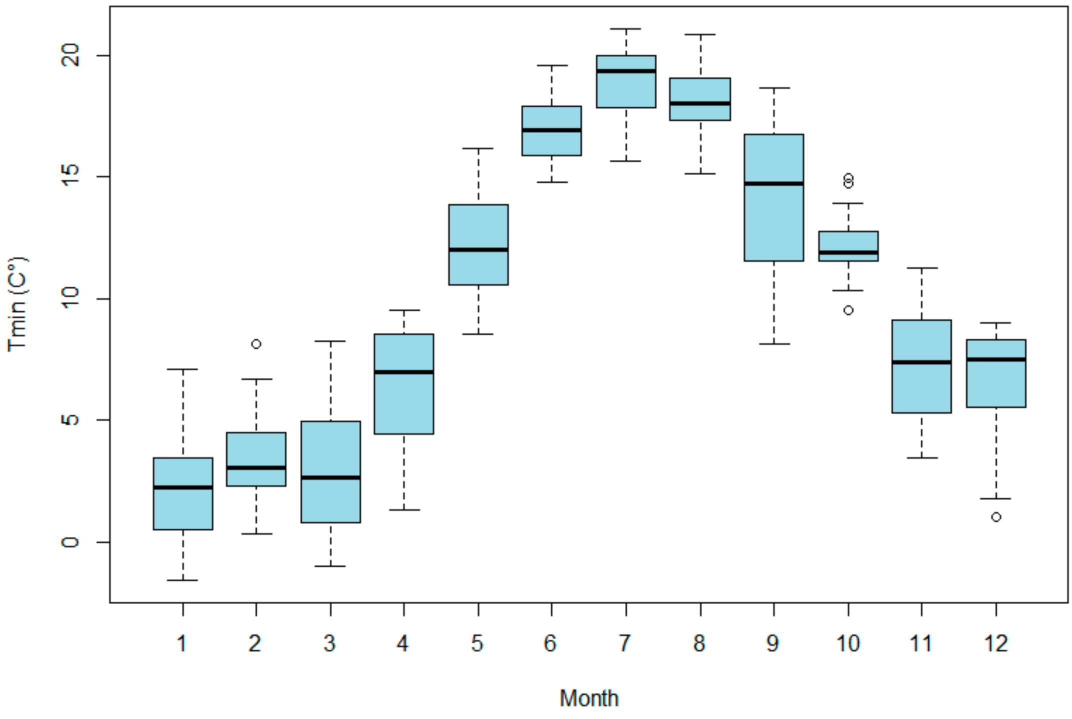

Figure S3. LST from Landsat8 (Summer 2022).

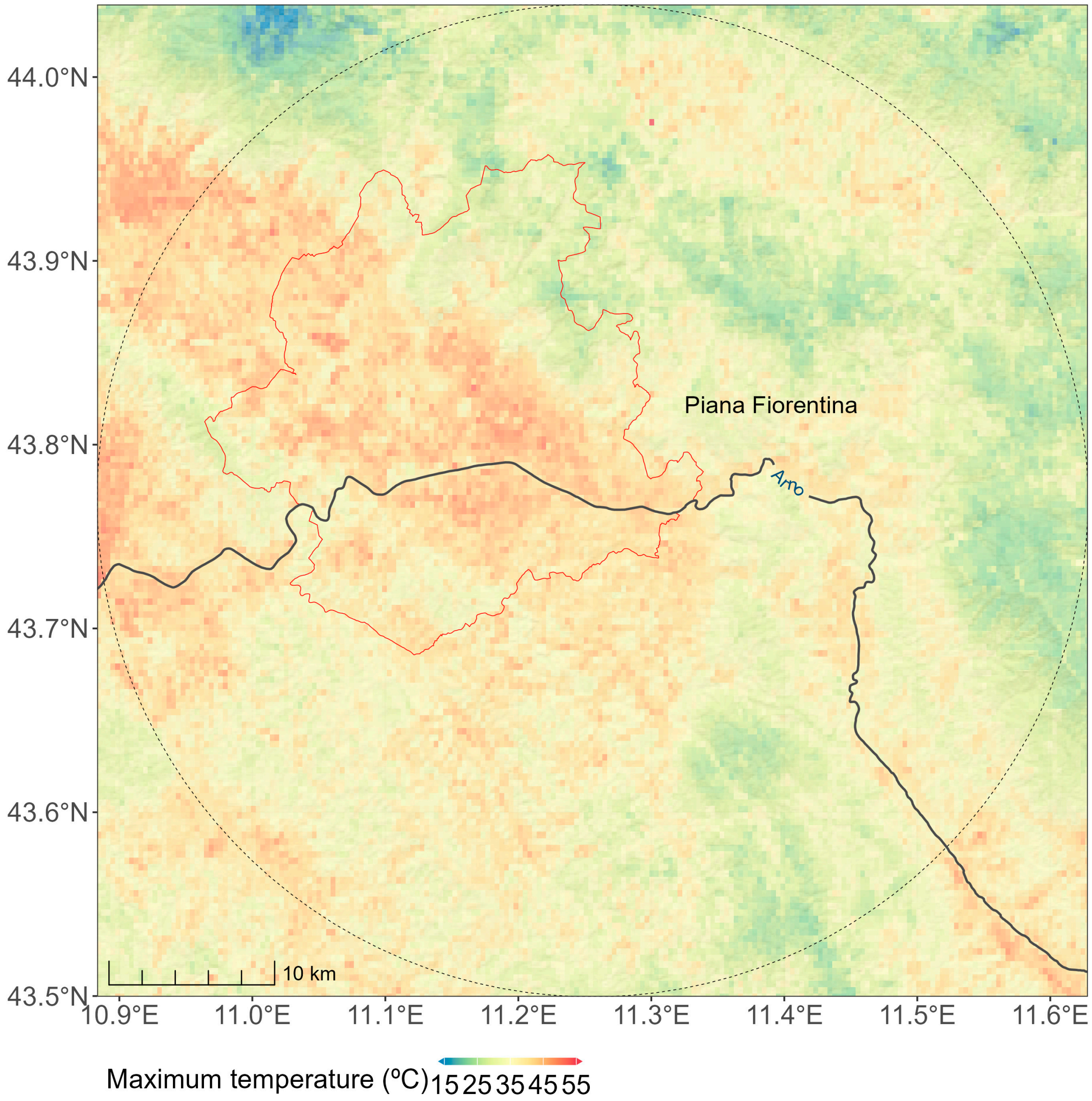

Table S1. Monitors stations in the Florence buffer (30km) area

ID	Municipality	Elevation (m)	Setting
TOS01000591	Montemignaio	745	Non Urban
TOS01000881	Castelfranco Piandiscò	395	Non Urban
TOS01000901	Reggello	980	Non Urban
TOS01001041	Rufina	955	Non Urban
TOS01001096	Firenze	84	Urban
TOS01001205	Prato	65	Urban
TOS01001215	Scandicci	42	Urban
TOS01001225	Sesto Fiorentino	33	Urban
TOS01001269	Agliana	40	Non Urban
TOS01004915	Montespertoli	117	Non Urban
TOS01004941	Empoli	25	Urban
TOS03001001	Vicchio	960	Non Urban
TOS03001097	Firenze	48	Urban
TOS03001099	Firenze	112	Urban
TOS03001135	Figline e Incisa Valdarno	500	Non Urban
TOS03001137	Bagno a Ripoli	382	Non Urban
TOS11000021	Barberino Tavarnelle	374	Non Urban
TOS11000022	Barberino Tavarnelle	430	Non Urban
TOS11000023	Greve in Chianti	536	Non Urban
TOS11000024	Pontassieve	230	Non Urban
TOS11000057	San Casciano in Val di Pesa	325	Non Urban
TOS11000071	Cerreto Guidi	70	Non Urban
TOS11000073	Greve in Chianti	254	Non Urban
TOS11000076	Carmignano	107	Non Urban
TOS11000089	Pelago	325	Non Urban
TOS11000093	Pistoia	125	Non Urban
TOS11000504	Empoli	90	Non Urban
TOS11000505	Montespertoli	198	Non Urban
